# Motivational drivers and Sense of Belonging: unpacking the persistence in Chinese Martial Arts practice among international practitioners

**DOI:** 10.3389/fpsyg.2024.1403327

**Published:** 2024-05-02

**Authors:** Xueying Cao, Hui Lyu

**Affiliations:** ^1^Faculty of Sports Science, Ningbo University, Ningbo, China; ^2^Ningbo Innovation Center, Zhejiang University, Ningbo, China

**Keywords:** Chinese martial arts (CMAs), persistence in practice, motivation, Sense of Belonging, questionnaire, structural equation modeling (SEM)

## Abstract

**Background:**

Chinese Martial Arts (CMAs) have garnered a global following, with their rich historical and cultural heritage transcending geographical and cultural differences, sparking profound interest among an international community. As an increasing number of non-Chinese individuals persist in practicing CMAs, investigating the motivations behind their continued participation has emerged as a compelling question. This study aims to delve deeper into the factors driving international practitioners to sustain their practice of CMAs, thereby broadening our understanding of the global resonance of CMAs.

**Methods:**

Employing Self-Determination Theory, 226 international CMAs practitioners completed the Physical Activity and Leisure Motivation Scale, Perceived Belonging Scale, and Persistence in Practicing CMAs Scale. SPSS 20.0 was utilized for conducting descriptive statistics, common method bias tests, and correlation analyses. Structural equation modeling was performed using AMOS 26.0.

**Results:**

Motivation for Practicing CMAs, comprised of enjoyment, mastery, physical condition, psychological condition, and appearance, has a positive impact on Persistence in Practicing CMAs (*β* = 0.297, *p* < 0.01). Sense of Belonging also positively affects Persistence in Practicing CMAs (*β* = 0.268, *p* < 0.01). The aforementioned variables account for 22.1% of the variance in Persistence in Practicing CMAs. Furthermore, Affiliation, Competition/Ego, and Others’ Expectations were found to have no significant correlation with Persistence in Practicing CMAs.

**Conclusion:**

The formation of persistence in the practice of CMAs among international practitioners is propelled by their ongoing desire for skill mastery, enjoyment, enhanced physical and mental health, body shape improvement, and a Sense of Belonging. The study reveals that a stronger motivation and Sense of Belonging significantly enhance their commitment to CMAs. Recommendations include that international instructors should center their teaching strategies around the practitioners, helping them to find joy in their practice, achieve skill mastery, and foster the development of physical, mental, and aesthetic qualities, alongside virtues and etiquette. Additionally, building a supportive CMAs community and cultivating a sense of ritual are essential. Such strategies are intended to reinforce practitioners’ self-affirmation and group identity, thus boosting their Sense of Belonging and encouraging their continued engagement in CMAs.

## 1 Introduction

The practice of Chinese Martial Arts (CMAs), with its rich historical and cultural heritage, extends far beyond the borders of China, captivating the interest of international practitioners worldwide (Farrer and Whalen-Bridge, 2011; Lau, 2022). Despite the geographical and cultural distances, a growing number of non-Chinese individuals are not only engaging in CMAs but are also showing a remarkable persistence in their practice (Jennings, 2010). This phenomenon raises intriguing questions about the motivational drivers behind their sustained interest they experience within this traditionally Chinese domain.

Martial arts represent a psychophysical cultural form deeply embedded in the traditions of hand-to-hand combat or weaponry, facilitating psychophysical enhancement and self-actualization through the training of fighting techniques (Cynarski, 2017). Recognizing the multifaceted nature of martial arts research, Cynarski (2017) introduced a comprehensive, interdisciplinary theoretical framework known as the General Theory of Fighting Arts (GTFA). This framework synthesizes three distinct perspectives: the Humanistic Theory of Martial Arts (HTMA), the Anthropology Theory of Martial Arts (AMA), and insights from Sports Science. Presently, research on CMAs has evolved across these three perspectives, encompassing cultural and philosophical discussions (Allen, 2014; An and Hong, 2018; Cibotaru, 2021), pedagogy and dissemination (Jia et al., 2022; Skowron-Markowska, 2022; Han et al., 2023; Ma and Jiang, 2023), as well as the health beneficial effects of CMAs practice (Gorgy et al., 2008; Fong et al., 2017; Zhang et al., 2023). While numerous studies have delved into why individuals participate in martial arts, findings depict a wide array of motivators shaped by varying disciplines and backgrounds of participants. Significantly, consistent key motivational themes have been identified across different martial arts disciplines. Specifically, Self-Determination Theory (SDT), with its intrinsic and extrinsic motivation categorization as outlined by Deci and Ryan (1985), offers a solid theoretical foundation for understanding exercise motivation. Morris and Rogers (2004) have organized eight motives under SDT into intrinsic (mastery and enjoyment) and extrinsic categories (the remaining six motives). Furthermore, they grouped the six extrinsic motives into body–mind (physical condition, psychological condition, appearance) and social motives (others’ expectations, affiliation, competition/ego) based on a second-order factor analysis. Mastery, along with the pursuit of physical and psychological health, stands out as pivotal motivators, as evidenced by martial arts practitioners, including those in Tai Chi, Taekwondo, and Karate, prioritizing these for their engagement (Morris and Han, 1991; Morris et al., 1995; Chowdhury, 2012; Witkowski et al., 2013; Molanorouzi et al., 2015; Zeng, 2019). Additionally, the significance of competition and ego in martial arts contexts has been highlighted, distinguishing participant motivations in their respective disciplines (Molanorouzi et al., 2015). Witkowski et al. (2013) reinforced this by noting judokas often cite winning prestigious competitions as a primary motivation. The search for enjoyment also emerges as a primary reason for young practitioners’ involvement in these disciplines (Zeng, 2019). Despite the initial insights into these motivational factors, the specific drivers that underpin the long-term engagement of practitioners in CMAs remain unclear. This identified gap underlines the imperative for an in-depth examination of the motivations propelling overseas practitioners to maintain their commitment to CMAs over an extended period.

**Figure 1:**
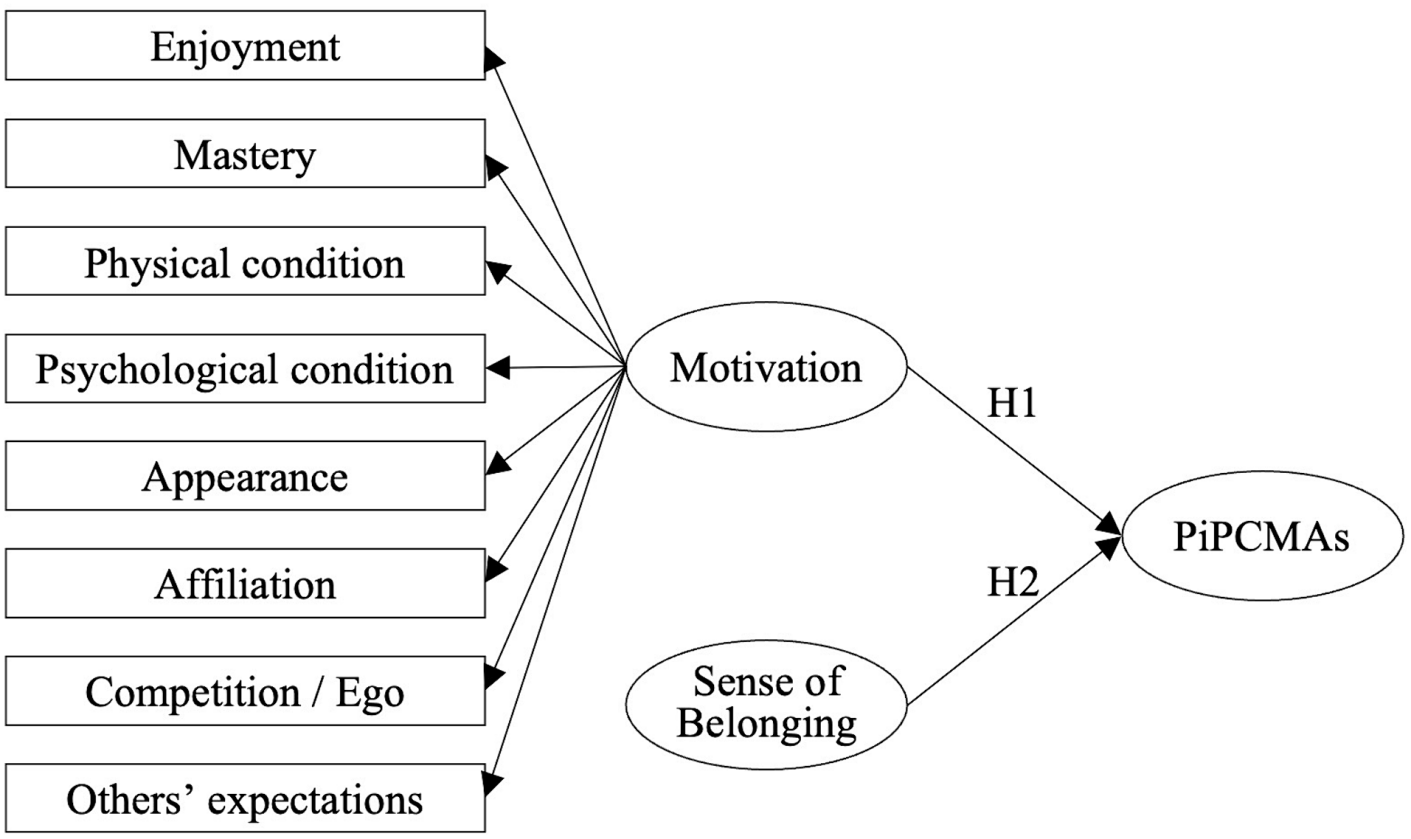
Research model. PiPCMA, Persistence in Practicing Chinese Martial Arts.

The relationship between motivation and the maintenance of long-term exercise is a significant topic of study in the fields of sports psychology and health promotion. Research has demonstrated that intrinsic motivations, such as enjoyment, are crucial in driving individuals to persist in their exercise routines (Ryan et al., 1997; Jiang and Wu, 2004; Chu et al., 2009; Zeng et al., 2013; Neys et al., 2014; Lee, 2018; Rodrigues et al., 2020). Furthermore, intrinsic motivation related to competence has also been identified as an important factor in exercise adherence (Ryan et al., 1997; Chu et al., 2009). However, the relationship between exercise persistence and motivations aimed at health, external appearance improvement, and social interaction presents varied results across different studies. For instance, Jiang and Wu (2004) and Chu et al. (2009) found that motivations related to physical and psychological health impact continuous exercise, while Ryan et al. (1997) observed that exercise adherence was unrelated to health motivations. Additionally, Ryan et al. (1997) associated social interaction with exercise persistence, and Jiang and Wu (2004) linked appearance (shaping the body, or gaining (losing) weight) with exercise adherence, whereas Chu et al. (2009) found no significant relationship between social, appearance motivations, and exercise persistence. Research suggests that different motivations are linked to various types of sports activities (Frederick and Ryan, 1993; Morris et al., 1995, 1996; Ryan et al., 1997; Molanorouzi et al., 2015), implying that the relationship between participation motivations and exercise adherence may vary across different sports. This study aims to explore the motivational mechanisms behind the persistence of practitioners of CMAs, drawing upon the aforementioned research to propose the following hypothesis (see Figure 1).

*H1*: Motivation for Practicing CMAs has a positive effect on Persistence in Practicing CMAs.

Incorporating the Self-Determination Theory (SDT), belongingness is identified as a fundamental psychological need, alongside autonomy and competence, that underpins human motivation and well-being (Deci and Ryan, 2000). The Sense of Belonging, or relatedness, is crucial for motivating self-determined behaviors, driving individuals to seek connections and nurture relationships, thereby fostering a sense of unity and mutual care within groups (Deci and Ryan, 1991). This theoretical perspective underscores the relational need for individuals to feel connected and valued within their social contexts. Partiková and Jennings (2018) argue that Kung Fu (referred to in this context as traditional CMAs) as Family – a way of thinking and feeling (and therefore acting) about Kung Fu in terms of family – provides a deep Sense of Belonging for people from a plethora of backgrounds. Kung Fu practitioners find belonging not only in their club but also within a wider group composed of distant cousins, recent seniors, forbearers, and ancestors. Fellow students are seen as siblings, with a responsibility to pass on the art to the next generation and nurture younger or incoming practitioners. Nardini and Scandurra (2021) suggest that practitioners pitted against each other in hand-to-hand clashes actually promote social exchange between opponents, offering them a common framework for collective identification. While these studies, employing qualitative methods, have emphasized the importance of belongingness in martial arts, no research has yet utilized quantitative methods to verify the impact of belongingness on practitioners’ long-term engagement in CMAs. Indeed, research in non-CMAs contexts has already found that group cohesiveness is related to individual adherence behavior (Carron et al., 1988). Additionally, Kopanidis (2023) identified the Sense of Belonging as one of the key factors influencing member retention and active participation. Based on this, the following hypothesis is proposed:

*H2*: Sense of Belonging has a positive effect on Persistence in Practicing CMAs.

In conclusion, applying Self-Determination Theory (SDT) to our research offers a valuable theoretical framework for an in-depth analysis of the complex motivations behind the long-term participation of international practitioners in CMAs. SDT’s multifaceted understanding of human motivation, which includes both intrinsic and extrinsic types (Deci and Ryan, 1985), allows us to explore more deeply the various motivations that drive individuals to continue practicing CMAs. Moreover, SDT emphasizes the importance of belongingness, autonomy, and competence (Deci and Ryan, 2000). In an international context, understanding how a Sense of Belonging influences practitioners’ commitment to persisting in CMAs is crucial for promoting the global dissemination of CMAs. Additionally, SDT research shows that when individuals’ behaviors are driven by intrinsic motivations, they are more likely to maintain those behaviors over time (Ryan et al., 1997). This insight is directly applicable to designing strategies to enhance long-term participation in CMAs and improving practitioners’ satisfaction and commitment. By integrating SDT into our study, our goal is not only to reveal the key psychological mechanisms underpinning the enduring commitment of international CMA practitioners but also to provide theoretical and practical insights for fostering sustained engagement in this rich cultural practice.

## 2 Materials and methods

### 2.1 Participants

In this study, we initiated data collection by employing Apify’s Google Maps Scraper service to gather information on 16,382 Chinese Martial Arts (CMAs) dojos, from which we identified 11,894 entries with website information. To refine our dataset for quality, we removed duplicates due to some dojos sharing the same website, resulting in 8,929 unique website URLs. We then utilized the Octoparse Web Scraping Tool to extract email addresses from the homepage of these websites, obtaining email addresses from 2,374 websites. A survey questionnaire, created via Google Forms, was distributed to these email addresses through a targeted “BCC” (blind carbon copy) email campaign. Additionally, to enhance our data collection, the survey was shared in various Facebook groups dedicated to Chinese martial arts, such as Chinese Martial Art & Kung-Fu Club, Traditional Chinese Martial Arts Community, among others, with prior consent from group administrators. This multi-faceted approach yielded 229 completed questionnaires from international CMAs practitioners. Following a rigorous screening process for patterned responses, inconsistencies, and duplicates, 226 questionnaires were validated for analysis, marking a valid response rate of 98.6%.

#### 2.1.1 Demographic characteristics

Table 1 presents the demographic characteristics of overseas practitioners of CMAs. The age distribution is mainly among four age groups: under 24 years (17.7%), 25–34 years (25.2%), 35–44 years (26.1%), and 45–54 years (22.1%). The sample is predominantly male (80.1%), with females only accounting for 18.6%. Regarding employment status, 59.3% are employed, 14.2% are self-employed, 15.9% are students, and 5.3% are retired. The level of education is generally high, with 27.4% holding a Bachelor’s degree, 22.6% a Master’s degree, 6.2% a Professional Degree, and 8% having some college credit but no degree, making up a total of 64.2% of the participants having attended university. The study sample includes a wide distribution of countries, predominantly English-speaking ones, such as the United States accounting for 24.3%. The survey being available only in English may influence the participation of non-English-speaking countries, which could also explain the higher level of education among the sample. The sample primarily consists of White individuals (64.6%), followed by Asians (15.0%). Non-Chinese participants account for 88.1%, and Chinese participants for 11.9%. Therefore, the results of this study may better explain the factors influencing the practice of CMAs among non-Chinese overseas. Respondents practicing for more than 10 years account for 41.1%, 6–10 years for 21.7%, 3–5 years for 21.7%, and 2 years or less for 15.5%, indicating that most of the collected samples have been exposed to CMAs for a significant period. 63.7% of the respondents learned CMAs from non-Chinese masters, 30.5% from Chinese masters, and only 5.8% have studied with both Chinese and non-Chinese masters. 64.2% of the respondents have watched CMAs competitions, only 30.5% have participated in CMAs competitions, 25.7% have come to China for CMAs exchanges, and 29.6% have participated in a CMAs tour (such as visiting the Shaolin Temple, Wudang Mountains, etc.).

**Table 1:** Demographic characteristics of the study group (*N* = 226).

Demographic Information	Distribution	Percentage (%)	Demographic Information	Distribution	Percentage (%)
Gender	Male	80.1	Ethnicity	White	64.6
	Female	18.6		Asian	15.0
	Prefer not to answer	1.3		Latino	10.2
Age	≤ 24	17.7		Other	10.2
	25–34	25.2	Religion	No Religion	44.7
	35–44	26.1		Christian	37.2
	45–54	22.1		Muslim	4.9
	≥ 55	8.5		Buddhist	3.1
	Prefer not to answer	0.4		Taoist	2.2
Country	United States	24.3		Other	6.2
	Brazil	14.2		Prefer not to answer	1.7
	Italy	9.7	Employment status	Employed	59.3
	Germany	6.2		A student	15.9
	United Kingdom	6.2		Self-employed	14.2
	Canada	4.4		Retired	5.3
	Netherlands	3.5		Other	5.3
	Turkey	3.5	Master is of Chinese descent	Yes	30.5
	Other	28.0		No	63.7
Education level	Bachelor’s degree	27.4		Both	5.8
	Master’s degree	22.6	Years of practice	< 1 year	7.1
	High school graduate	15.9		1–2 years	8.4
	Some college credit, no degree	8.0		3–5 years	21.7
	Professional degree	6.2		6–10 years	21.7
	Other	19.9		> 10 years	41.1
Martial status	Single	40.7	Watched CMAs competitions	Yes	64.2
	Married	44.3		No	35.8
	Engaged	8.0	Participated in CMAs competitions	Yes	30.5
	Divorced	4.4		No	69.5
	Widowed	0.9	Participated in a CMAs tour	Yes	29.6
	Prefer not to answer	1.7		No	70.4
Ethnic Chinese	Yes	11.9	Participated in CMAs exchange in China	Yes	25.7
	No	88.1		No	74.3

#### 2.1.2 CMAs content selection

The survey results indicate a broad array of preferences among practitioners for traditional Chinese Martial Arts (CMAs), with certain styles enjoying particular popularity. Notably, more than 30% of respondents favor Wing Chun (35.4%) and Yang-style Tai Chi (33.6%). Styles preferred by over 20% include Qigong (27.9%) and Shaolin Kung Fu (24.8%), while those chosen by over 10% encompass Xing Yi Quan (19.9%), Baguazhang (17.7%), Chen-style Tai Chi (16.4%), Hung Ga (11.5%), Choy Li Fut (11.1%), Praying Mantis (10.6%), and Qinna (10.6%). Beyond these, a wide range of other styles are practiced by respondents, such as Bajiquan, Wudang Martial Arts, Hakka Kung Fu, Shuai Jiao, Eagle Claw, White Crane, Bak Mei, Zhou Family Praying Mantis, Pi Gua Quan, Cha Quan, Xin Yi Liu He Quan, Emei Fire Dragon Quan, Pi Gua Zhang, Short Weaponry, Lai Tung Pai, and Tith Ngaw Pai, showcasing the rich diversity of CMAs globally. Furthermore, 32.3% of respondents exclusively practice CMAs, yet a significant proportion also engage in other martial arts, including Karate (29.2%), Taekwondo (17.7%), Judo (17.3%), Brazilian Jiu-Jitsu (16.4%), Boxing (15.5%), and Muay Thai (12.4%). This variety in martial arts practices among CMA practitioners abroad highlights the global appeal and diversity of martial arts disciplines.

The essence of CMAs is combat skill, with 90.3% of respondents believing they have learned the application of techniques and improved their fighting skills through studying CMAs. 82.7% feel they have learned martial virtues (Wu De) such as respect, self-discipline, perseverance, commitment, and trust. 81.9% understand the historical background and lineage of the CMAs they studied, 71.7% are aware of the cultural and philosophical foundations of CMAs, such as Yin-Yang and the Bagua, 54.4% have learned etiquette like the fist and palm salute, and only 13.7% are familiar with the master-apprentice ceremony. Additionally, some respondents noted that “language is also an important aspect gained during the learning process of CMAs, having learned to name movements in Cantonese, as well as terms for master, grandmaster, male and female fellow disciples, and counting in Cantonese.”

### 2.2 Instruments

This study incorporated three scales: Motivation for Practicing CMAs Scale, Perceived Belonging Scale and Persistence in Practicing CMAs Scale. To ensure the reliability and validity of the measurement tools, this research primarily utilized scales that have been previously employed in studies, which were then modified according to the research objectives to serve as empirical tools.

- Motivation for Practicing CMAs Scale. Motivation is an intrinsic force influencing behavior. The PALMS (Physical Activity and Leisure Motivation Scale) is a comprehensive tool based on Self-Determination Theory that measures motivation for participating in physical sports activities (Morris and Rogers, 2004). This scale is a condensed version of the 73-item REMM (Recreational Exercise Motivation Measure), categorizing motivation into eight dimensions for participating in physical activities (Rogers, 2000). These dimensions include Enjoyment, Mastery, Physical condition, Psychological condition, Appearance, Affiliation, Competition/Ego, and Others’ expectations. Enjoyment motivation is due to finding it fun, enjoyable, and happiness-inducing; Mastery motivation is due to the desire to acquire new skills and improve abilities; Physical condition motivation is for physical health and a robust physique. Psychological condition motivation is for stress relief and relaxation; Appearance motivation is to improve body shape and appearance; Affiliation motivation is for making new friends or spending time with friends. Competition/Ego motivation is to surpass or exceed others; Others’ expectations motivation is due to others expecting you to do so. The scale consists of 40 items, with alpha coefficients of all eight dimensions being 0.78 or higher, indicating high structural validity as confirmed through structural equation modeling (Molanorouzi et al., 2014). Items are measured on a Likert 5-point scale, ranging from 1 (Strongly disagree) to 5 (Strongly agree).
- Perceived Belonging Scale. This scale is based on Self-Determination Theory and uses 11 items to measure the perceived Sense of Belonging. The scale’s Cronbach’s alpha coefficients are all above 0.70, and its construct validity has been verified through Structural Equation Modeling, showing a high level of fit (Allen, 2006). Items are measured using a Likert 7-point scale, ranging from 1 (Disagree strongly) to 7 (Agree strongly).
- Persistence in Practicing CMAs Scale. Derived from exercise adherence, which refers to the individual’s tendency to demonstrate enduring, continuous, or effortful behavior during physical exercise (Wang et al., 2016). This study employs the persistence scale by Liu et al. (2011), which has an alpha coefficient of 0.85 and good fit. After revising to suit Persistence in Practicing CMAs, the scale was translated into English through iterative back-translation by bilingual translators (Brislin, 1980). Items are measured using a Likert 5-point scale, with options ranging from 1 (Strongly disagree) to 5 (Strongly agree).

### 2.3 Analysis

The study utilized SPSS 20.0 and the Structural Equation Modeling (SEM) software AMOS 26.0 to conduct empirical analysis following these steps: First, employing a single-factor method for common method bias test; Second, using confirmatory factor analysis to test reliability and validity as well as the fit of the measurement model; Third, exploring the relationships between variables through correlation analysis; Fourth, assessing the overall fit of the structural model; Fifth, revising and interpreting the results of the model fit.

Additionally, the data underwent the following processes:

- Scale conversion. As the majority of scales used in this study were 7-point scales, the 5-point scale, was uniformly converted to 7-point scales for data analysis. The conversion formula is: $Y=\left(B-A\right)\times \frac{x-a}{b-a}+A$ , where Y is the function of the converted scale, X is the function of the scale used in the original questionnaire, a and b are the minimum and maximum values of the original scale, and A and B are the minimum and maximum values of the converted scale, respectively.
- Item parceling. SEM analysis typically requires a sample to observed variable ratio of at least 10:1 (Thompson, 2000). Given the difficulty of obtaining overseas sample data and the small sample size and complex model of this study, to meet the sample size requirements for SEM analysis, this study simplified the model by item parceling of first-order latent variables in the second-order model of Motivation for Practicing CMAs, based on the reliability and validity testing of the scales’ various latent variables.

## 3 Results

### 3.1 Test for common method bias

Given that all data were self-reported by CMAs practitioners, the study first employed Harman’s single-factor test to examine common method bias. The exploratory factor analysis with rotation identified nine factors with eigenvalues greater than 1, where the largest factor accounted for 24.067% of the variance. These results are in line with the criteria proposed by Podsakoff et al. (2003), where more than one factor with eigenvalues greater than 1 and the largest factor’s variance explanation being less than 40% indicate that severe common method bias is not present in this study.

### 3.2 Reliability and validity test and analysis of the fit of the measurement model

This study analyses the correspondence between measurement factors and items through confirmatory factor analysis (CFA) (see Table 2).

- Measurement model fit. Items within each latent variable with standardized factor loadings below 0.4 were deleted (Hair et al., 2014); model fit indices were checked, and items causing excessively high chi-square values due to residual correlations, indicating item similarity, were removed (Landis et al., 2009). Ultimately, items a_4, b_1, b_5, c_1, d_3, d_4, e_2, f_3, g_5, h_1, and h_3 from the Motivation for Practicing CMAs scale, items 1, 3, 8, 9, and 10 from the Perceived Belonging Scale, and items 1 and 6 from the Persistence in Practicing CMAs Scale were deleted, thereby achieving a better model fit for each factor (Hair et al., 2014). Factors not annotated with fit indices in the table are due to having only three items, constituting a just-identified model, where the number of data points matches the number of parameters to be estimated in the model, resulting in zero degrees of freedom, also known as a saturated model.
- Internal consistency coefficient (*α*). This value is a commonly used index for testing reliability, with the formula: $\alpha =\frac{K}{K-1}\left(1-\frac{\sum S_{i}^{2}}{S^{2}}\right)$ , where K is the number of items in the scale, $\Sigma S_{i}^{2}$ is the total variance of the scale items, and $S^{2}$ is the variance of the total score of the scale items. The α coefficient ranges from 0 to 1, with DeVellis (2017) suggesting that values between 0.65 and 0.70 are the minimum acceptable; values between 0.70 and 0.80 are quite good; values between 0.80 and 0.90 are very good. Almost all factors in this study had alpha coefficients above 0.70, indicating good internal consistency, with only one factor (Mastery) having an alpha value of 0.687, which is still within an acceptable range.
- Convergent validity is represented by the average variance extracted (AVE), which can be calculated using the formula: $AVE=\frac{\left(\sum \lambda^{2}\right)}{\left(\left(\sum \lambda^{2}\right)+\sum \theta \right)}$ , where *λ* represents the standardized factor loadings of the observed variables on the latent variable, and θ represents the error variance of the indicator variables. AVE reflects the extent to which a latent variable construct can explain the variance of its indicator variables. Higher AVE values indicate higher reliability and convergent validity of the construct. Fornell and Larcker (1981b) consider values between 0.36 to 0.5 as the minimum acceptable, and values above 0.5 as ideal. Most factors in this study had AVE values above 0.5, indicating good convergent validity. Only three factors (Enjoyment, Mastery, PiPCMAs) had AVE values below 0.5 but ≥0.36, which is still within an acceptable range.
- Composite Reliability (CR). This value can be calculated using the formula: $CR=\frac{{\left(\sum \lambda \right)}^{2}}{\left({\left(\sum \lambda \right)}^{2}+\sum \theta \right)}$ , where λ represents the standardized factor loadings of the observed variables on the latent variable, and θ represents the error variance of the indicator variables. CR indicates whether all items within each latent variable consistently explain that latent variable. Fornell and Larcker (1981a) suggest that a CR value above 0.6 indicates good composite reliability. All latent variables in this study had CR values above 0.6, indicating good composite reliability.

**Table 2:** Summary of confirmatory factor analysis for each factor in the research model.

Factor	Item	Model parameter estimates				Convergent validity				Goodness-of-fit indexes					
		UFL	S.E.	*t*	*P*	SFL	SMC	C.R.	AVE	χ^2^	DF	χ^2^/df	GFI	AGFI	RMSEA
Enjoyment (*α* = 0.726)	Q14a_1	1.000				0.502	0.252	0.740	0.426	0.257	2	0.129	0.999	0.997	0.000
	Q14a_2	1.323	0.243	5.447	***	0.515	0.265								
	Q14a_3	2.687	0.418	6.432	***	0.820	0.672								
	Q14a_5	2.615	0.406	6.440	***	0.718	0.516								
Mastery (*α* = 0.687)	Q14b_2	1.000				0.584	0.341	0.695	0.434	0.000	0	–	–	–	–
	Q14b_3	1.526	0.252	6.044	***	0.660	0.436								
	Q14b_4	1.637	0.279	5.871	***	0.725	0.526								
Physical condition (*α* = 0.752)	Q14d_1	1.000				0.821	0.674	0.789	0.560	0.000	0	–	–	–	–
	Q14d_2	1.328	0.151	8.805	***	0.815	0.664								
	Q14d_5	1.122	0.143	7.867	***	0.584	0.341								
Psychological condition (*α* = 0.854)	Q14g_1	1.000				0.659	0.434	0.858	0.605	0.184	2	0.092	1.000	0.998	0.000
	Q14g_2	1.445	0.146	9.925	***	0.785	0.616								
	Q14g_3	1.584	0.164	9.670	***	0.759	0.576								
	Q14g_4	1.725	0.162	10.616	***	0.891	0.794								
Appearance (*α* = 0.896)	Q14f_1	1.000				0.797	0.635	0.896	0.684	1.024	2	0.512	0.998	0.989	0.000
	Q14f_2	1.121	0.080	14.021	***	0.857	0.734								
	Q14f_4	1.129	0.086	13.110	***	0.809	0.654								
	Q14f_5	1.173	0.085	13.789	***	0.844	0.712								
Affiliation (*α* = 0.834)	Q14c_2	1.000				0.656	0.430	0.836	0.563	4.058	2	2.029	0.991	0.955	0.068
	Q14c_3	1.304	0.136	9.613	***	0.801	0.642								
	Q14c_4	1.234	0.141	8.743	***	0.700	0.490								
	Q14c_5	1.300	0.133	9.773	***	0.831	0.691								
Competition/Ego (*α* = 0.832)	Q14e_1	1.000				0.518	0.268	0.837	0.570	0.629	2	0.315	0.999	0.993	0.000
	Q14e_3	1.813	0.240	7.563	***	0.802	0.643								
	Q14e_4	1.830	0.241	7.595	***	0.811	0.658								
	Q14e_5	1.949	0.253	7.690	***	0.843	0.711								
Others’ expectations (*α* = 0.787)	Q14h_2	1.000				0.652	0.425	0.793	0.565	0.000	0	–	–	–	–
	Q14h_4	1.305	0.157	8.301	***	0.868	0.753								
	Q14h_5	1.093	0.127	8.594	***	0.718	0.516								
Sense of Belonging (*α* = 0.902)	Q13_2	1.000				0.771	0.594	0.906	0.619	16.381	9	1.820	0.976	0.944	0.060
	Q13_4	1.152	0.098	11.793	***	0.752	0.566								
	Q13_5	1.221	0.085	14.407	***	0.892	0.796								
	Q13_6	0.816	0.067	12.191	***	0.774	0.599								
	Q13_7	0.806	0.077	10.481	***	0.680	0.462								
	Q13_11	1.007	0.075	13.355	***	0.835	0.697								
PiPCMAs (*α* = 0.733)	Q8_2	1.000				0.593	0.352	0.743	0.429	0.040	2	0.020	1.000	1.000	0.000
	Q8_3	1.106	0.156	7.108	***	0.645	0.416								
	Q8_4	1.699	0.232	7.309	***	0.839	0.704								
	Q8_5	0.961	0.164	5.865	***	0.493	0.243								

UFL, Unstandardized Factor Loadings; S.E., Standard Error; SFL, Standardized Factor Loadings; SMC, Squared Multiple Correlations; C.R., Composite Reliability; AVE, Average Variance Extracted; df, Degrees of Freedom; GFI, Goodness of Fit Index; AGFI, Adjusted Goodness of Fit Index; RMSEA, Root Mean Square Error of Approximation; PiPCMA, Persistence in Practicing Chinese Martial Arts; *** *p* < 0.001.

### 3.3 Correlation analysis of Motivation for Practicing CMAs, Sense of Belonging, and Persistence in Practicing CMAs

Table 3 presents the descriptive statistics and correlation coefficients for Motivation for Practicing CMAs, Sense of Belonging, and Persistence in Practicing CMAs. Correlation coefficients with statistical significance related to Persistence in Practicing CMAs are highlighted in bold, with M ± SD denoting mean ± standard deviation. The bold italic numbers on the diagonal are the square roots of the Average Variance Extracted (AVE) for each variable. The results indicate that there is a significant positive correlation between Persistence in Practicing CMAs and Sense of Belonging (*r* = 0.347, *p* < 0.01). Additionally, Persistence in Practicing CMAs is positively correlated with aspects of Motivation for Practicing CMAs, including Enjoyment (*r* = 0.306, *p* < 0.01), Mastery (*r* = 0.422, *p* < 0.01), Physical Condition (*r* = 0.345, *p* < 0.01), Psychological Condition (*r* = 0.295, *p* < 0.01), and Appearance (*r* = 0.150, *p* < 0.01). However, there are no significant correlations between Persistence in Practicing CMAs and other motivational aspects such as Affiliation, Competition/Ego, and Others’ expectations. Furthermore, comparing the square root of each variable’s AVE with the correlation coefficients between that variable and others reveals that the square root of the AVE for each variable is greater than its correlation coefficients with other variables, indicating discriminant validity among the variables in this study.

**Table 3:** Descriptive statistics and correlation coefficients for factors (*N* = 226).

	M ± SD	(1)	(2)	(3)	(4)	(5)	(6)	(7)	(8)	(9)	(10)
(1) PiPCMAs	5.649 ± 1.105	***0.655***	–	–	–	–	–	–	–	–	–
(2) Sense of Belonging	6.256 ± 0.744	0.347**	***0.787***	–	–	–	–	–	–	–	–
(3) Enjoyment	6.343 ± 0.692	0.306**	0.362**	***0.653***	–	–	–	–	–	–	–
(4) Mastery	6.079 ± 0.795	0.422**	0.346**	0.591**	***0.659***	–	–	–	–	–	–
(5) Physical condition	6.155 ± 0.810	0.345**	0.328**	0.506**	0.583**	***0.748***	–	–	–	–	–
(6) Psychological condition	5.741 ± 1.111	0.295**	0.277**	0.401**	0.394**	0.576**	***0.778***	–	–	–	–
(7) Appearance	4.507 ± 1.673	0.150*	0.172**	0.313**	0.451**	0.550**	0.442**	***0.827***	–	–	–
(8) Affiliation	4.405 ± 1.487	0.081	0.307**	0.269**	0.281**	0.273**	0.411**	0.334**	***0.750***	–	–
(9) Competition/Ego	3.149 ± 1.586	0.076	0.169*	0.193**	0.356**	0.241**	0.256**	0.524**	0.406**	***0.755***	
(10) Others’ expectations	1.867 ± 1.241	−0.062	−0.002	−0.006	−0.041	0.118	0.162*	0.299**	0.391**	0.567**	***0.752***

The bold numbers on the diagonal are the square roots of the AVE for that variable; PiPCMAs, Persistence in Practicing Chinese Martial Arts; * *p* < 0.05; ** *p* < 0.01.

### 3.4 Structural model test of the persistence behavior formation mechanism in practicing CMAs

#### 3.4.1 Testing the second-order model of Motivation for Practicing CMAs

**Figure 2:**
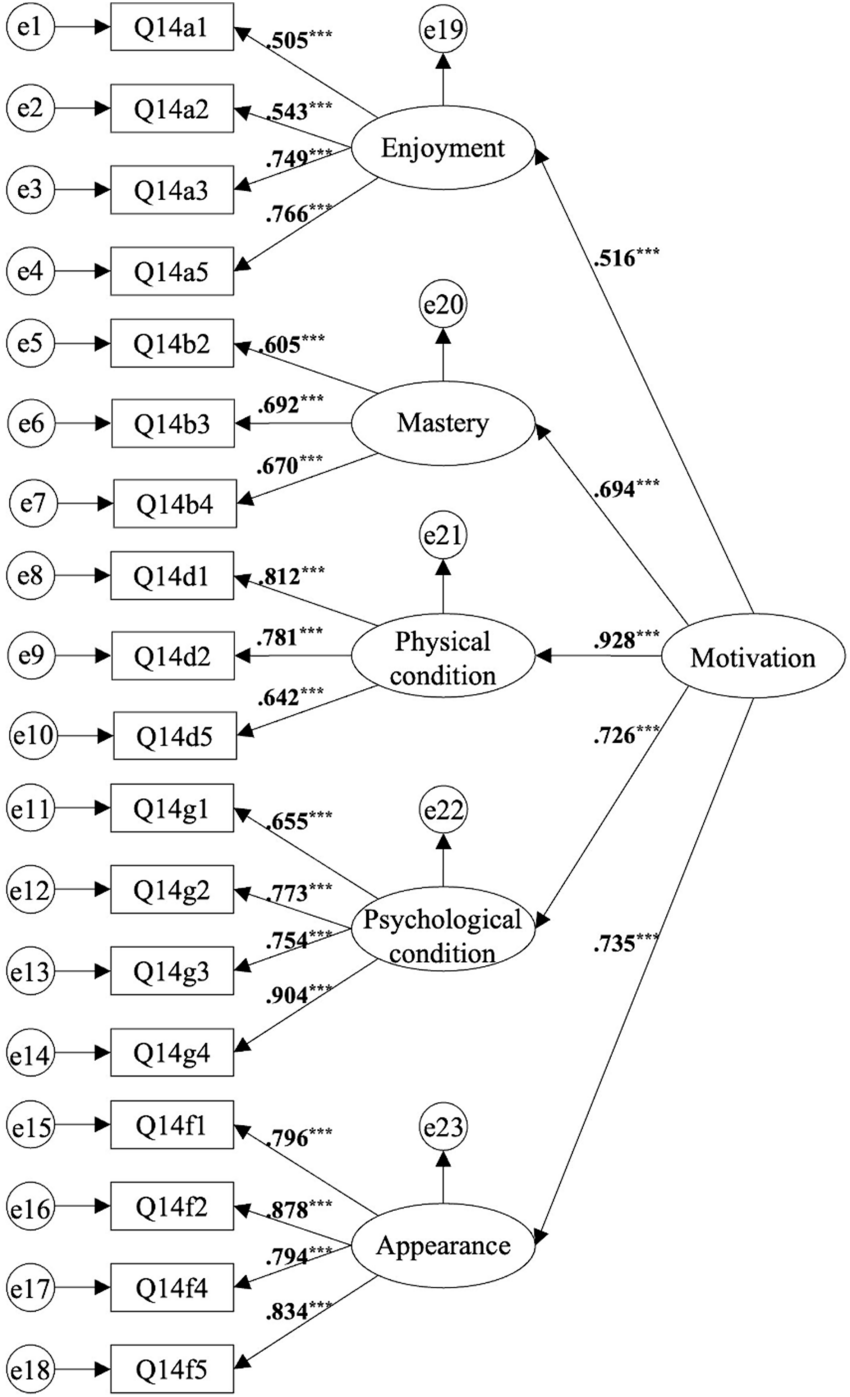
Second-order model of Motivation for Practicing Chinese Martial Arts; *** *p* < 0.001.

Correlational analysis among variables revealed (1) Within Motivation for Practicing CMAs, Affiliation, Competition/Ego, and Others’ expectations showed no significant correlation with Persistence in Practicing CMAs, while the other five dimensions of motivation (Enjoyment, Mastery, Physical Condition, Psychological Condition, and Appearance) were all related to Persistence in Practicing CMAs; (2) The correlations among the five motivations for practicing CMAs (Enjoyment, Mastery, Physical Condition, Psychological Condition, and Appearance) were generally moderate (*r* > 0.4) (see Table 3). In SEM, when first-order factors are moderately to highly correlated and all influenced by a higher-order latent trait, a second-order confirmatory factor analysis can be performed (Wu, 2010). Therefore, the study considered Enjoyment, Mastery, Physical Condition, Psychological Condition, and Appearance as first-order factors and Motivation for Practicing CMAs as a second-order factor to test the fit of the Motivation for Practicing CMAs second-order model (see Figure 2).

First, testing whether the second-order model can explain the first-order model. Marsh and Hocevar (1985) argue that a target coefficient (the chi-square value of the first-order factors divided by the chi-square value of the second-order model) closer to 1 indicates greater accuracy of the second-order model. By calculating the target coefficient, it was found that the target coefficient for Motivation for Practicing CMAs is 0.918 (253.067/275.551), indicating excellent adaptability of the second-order CFA index (see Table 4).

**Table 4:** Goodness-of-fit indexes for alternative models of the second-order Motivation for Practicing Chinese Martial Arts (*N* = 226).

	*χ* ^2^	df	*χ*^2^/df	GFI	AGFI	CFI	RMSEA
0. Null model	2027.024	153	13.249	0.323	0.243	0.000	0.233
1. 1 First-order factor	830.427	135	6.151	0.646	0.551	0.629	0.151
2. 5 First-order factors (Uncorrelated)	573.189	135	4.246	0.760	0.695	0.766	0.120
3. 5 First-order factors (Correlated)	**253.067**	125	2.025	0.894	0.854	0.932	0.067
4. 1 Second-order factor	**275.551**	130	2.120	0.885	0.849	0.922	0.071
Reference value	Smaller is better	Larger is better	< 3	> 0.9	> 0.9	> 0.9	< 0.08

df, Degrees of Freedom; GFI, Goodness of Fit Index; AGFI, Adjusted Goodness of Fit Index; CFI, Comparative Fit Index; RMSEA, Root Mean Square Error of Approximation.

Bold values are key indicators for calculating the target coefficient.

Second, testing the fit of the second-order model. The RMSEA of the second-order model for Motivation for Practicing CMAs is 0.071, GFI = 0.885, CFI = 0.922, *χ*^2^/df = 2.120, indicating that the model fits well.

Third, testing the convergent validity and construct reliability of the second-order model. Upon testing, the Average Variance Extracted (AVE) of the second-order model for Motivation for Practicing CMAs is 0.535, greater than 0.5, indicating good convergent validity; the Construct Reliability (CR) is 0.848, significantly higher than 0.6, indicating good construct reliability (see Table 5).

**Table 5:** Confirmatory factor analysis for the second-order model of Motivation for Practicing Chinese Martial Arts.

Second-order factor	First-order factors	Model parameter estimates				Convergent validity			
		UFL	S.E.	*t*	*p*	SFL	SMC	C.R.	AVE
Motivation (*α* = 0.763)	Enjoyment	1.000				0.516	0.266	0.848	0.535
	Mastery	2.315	0.554	4.180	***	0.694	0.482		
	Physical condition	4.310	0.888	4.853	***	0.928	0.861		
	Psychological condition	3.211	0.716	4.482	***	0.726	0.527		
	Appearance	6.223	1.328	4.685	***	0.735	0.540		

UFL, Unstandardized Factor Loadings; S.E., Standard Error; SFL, Standardized Factor Loadings; SMC, Squared Multiple Correlations; C.R., Composite Reliability; AVE, Average Variance Extracted; *** *p* < 0.001.

In conclusion, the second-order model of Motivation for Practicing CMAs is acceptable, meaning it can effectively explain the constructs of the first-order factors.

#### 3.4.2 Testing the formation mechanism model of Persistence in Practicing CMAs

Motivation for Practicing CMAs and Sense of Belonging were treated as exogenous variables, while Persistence in Practicing CMAs was treated as an endogenous variable, and the model was fitted using the Maximum Likelihood (ML) estimation method.

**Figure 3:**
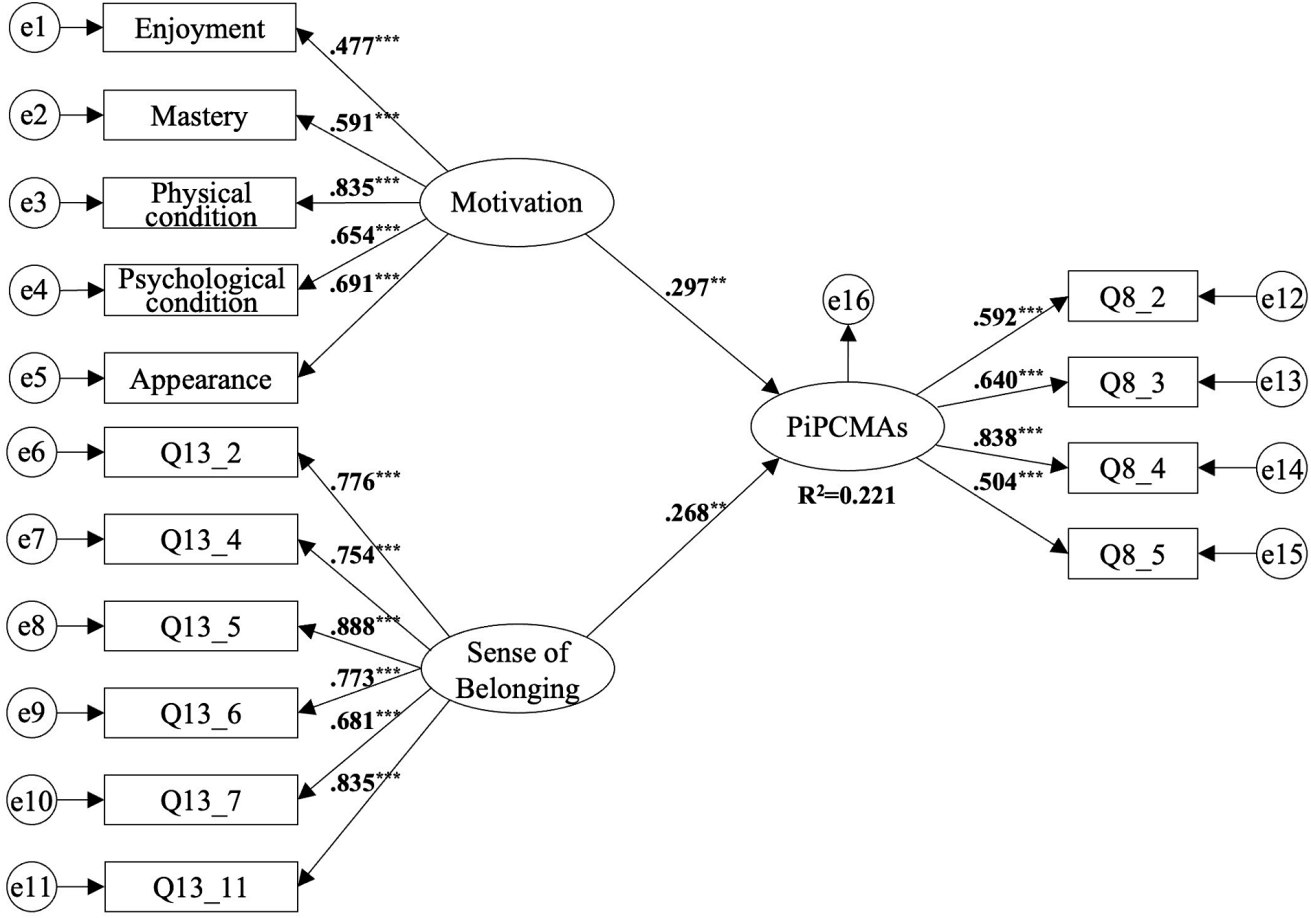
Research model path diagram. PiPCMAs, Persistence in Practicing Chinese Martial Arts; ** *p* < 0.01; *** *p* < 0.001.

- Overall Model Fit Test. The model fit indices were examined from three aspects: absolute fit indexes, incremental fit indexes, and parsimony fit indexes. Among these, the absolute fit indexes showed χ^2^ = 186.790, GFI = 0.899, close to 0.9, and RMSEA = 0.071, less than 0.08; the incremental fit indices IFI = 0.931, TLI = 0.916, and CFI = 0.931, all greater than 0.90; the parsimony fit index χ^2^/df = 2.147, satisfying the criterion of being less than 3, indicating a good overall fit of the structural equation model.
- Model Path Analysis and Hypothesis Testing. Figure 3 presents the structural model of the persistence behavior formation mechanism in overseas CMAs practitioners. The results indicate that Motivation for Practicing CMAs, composed of Enjoyment, Mastery, Physical Condition, Psychological Condition, and Appearance, has a significant positive impact on Persistence in Practicing CMAs (*β* = 0.297, *b* = 0.489, *t* = 3.062, *p* < 0.01), supporting H1; Sense of Belonging also significantly positively affects Persistence in Practicing CMAs (*β* = 0.268, *b* = 0.189, *t* = 3.141, *p* < 0.01), supporting H2; the model explains 22.1% of the variance in Persistence in Practicing CMAs (see Table 6).

**Table 6:** Path analysis of the research model.

Paths between latent factors	*b*	S.E.	*t*	*β*	*p*	Corresponding hypothesis	Test result
PiPCMAs ← Motivation	0.489	0.160	3.062	0.297	0.002	H1	✓
PiPCMAs ← Sense of Belonging	0.189	0.060	3.141	0.268	0.002	H2	✓

*b*, Unstandardized Path Coefficients, represents the direct effect of one variable on another, measured in the original units of the variables; S.E., Standard Error; *β*, Standardized Path Coefficients, indicates the strength and direction of the relationship between variables, measured in standard deviation units, facilitating comparison across different paths in the model; PiPCMAs, Persistence in Practicing Chinese Martial Arts.

## 4 Discussion

This study, grounded in Self-Determination Theory and exploring from a motivational viewpoint, conducted an empirical investigation into the mechanisms behind the persistence behavior of overseas practitioners in CMAs. Our findings reveal that: (1) The motivation to practice, encompassed by Enjoyment, Mastery, Physical Condition, Psychological Condition, and Appearance, exerts a significant positive impact on Persistence in Practicing CMAs; (2) A Sense of Belonging significantly positively affects Persistence in Practicing CMAs; (3) Motivational factors such as Affiliation, Competition/Ego, and Others’ Expectations do not exhibit a significant correlation with Persistence in Practicing CMAs.

### 4.1 Conceptual explanation of Persistence in Practicing CMAs

Persistence in Practicing CMAs denotes the level of sustained effort exhibited by practitioners when faced with challenges and obstacles during their training. The evaluation of this concept through standardized factor loadings of survey items (see Table 2) offers insightful revelations about persistence. The findings, ordered by their factor loadings, reveal a hierarchy of persistent elements: the highest loading is observed for the statement “Living without Chinese Martial Arts would be difficult for me” (0.839), underscoring the integral role that CMAs play in the lives of practitioners. This sentiment reflects a deep emotional bond with the practice, suggesting that the absence of CMAs would significantly impact their lifestyle and well-being. Following this, the item “I would feel disheartened if I had to stop practicing Chinese martial arts” (0.645) highlights the emotional investment practitioners place in their training, where the prospect of discontinuation evokes a sense of loss. This emotional investment is pivotal in fostering continued engagement with CMAs, even in the face of challenges. The statement “Despite my limited time, I’ve never stopped practicing Chinese martial arts” (0.593) illustrates the practitioners’ commitment and effective time management, indicating that they prioritize CMAs practice despite competing demands on their time. Lastly, the item “Even though there are difficulties in practicing Chinese martial arts, I am willing to persist in practicing it” (0.493), despite its relatively lower factor loading, underscores the resilience and determination among practitioners. Drawing on the qualitative sociological research by Jennings (2010), which posits that the practice of CMAs among British practitioners transcends mere physical training to encompass broader socio-cultural and psychological commitments, this resilience is further understood as a testament to the practitioners’ dedication, where challenges and obstacles do not deter their commitment to CMAs.

In sum, these insights reveal that a blend of emotional attachment, commitment to practice, and resilience against challenges contributes to the high persistence observed among CMA practitioners. Such persistence is not merely a function of habit but a reflection of a profound connection to the art, underscoring its significance in their lives and identities. This analysis not only elucidates the factors underpinning persistence in CMAs practice but also highlights the multifaceted relationship practitioners have with their art, characterized by emotional depth, dedication, and resilience. These insights are pivotal for comprehending and enhancing the global dissemination of CMAs and ensuring its continuous practice among overseas practitioners.

### 4.2 Explanation of the formation mechanism of Persistence in Practicing CMAs

#### 4.2.1 The impact of motivation on Persistence in Practicing CMAs

Morris’s classification divides eight motivational dimensions into three categories: intrinsic motivation (Enjoyment, Mastery), extrinsic psychophysical motivation (Physical Condition, Psychological Condition, Appearance), and extrinsic social motivation (Affiliation, Competition/Ego, Others’ Expectations) (Morris and Rogers, 2004).

The study’s results demonstrate a significant positive impact of the composite motivation for practicing CMAs - which includes Enjoyment, Mastery, Physical Condition, Psychological Condition, and Appearance - on the persistence of practice (*β* = 0.297, *p* < 0.01). Notably, the order of influence, based on standardized factor loadings, is Physical Condition (0.835), followed by Appearance (0.691), Psychological Condition (0.654), Mastery (0.591), and Enjoyment (0.477). Conversely, extrinsic social motivations such as Affiliation, Competition/Ego, and Others’ Expectations show no substantial correlation with the persistence in CMAs practice. This elucidation suggests that while intrinsic and psychophysical motivations significantly foster continued engagement with CMAs, external social pressures and expectations do not markedly affect practitioners’ dedication. This insight underscores the complex interplay of personal fulfillment and physical well-being in sustaining CMAs practice, rather than external validation or social affiliations.

##### 4.2.1.1 Intrinsic motivation

The intrinsic motivations for engaging in CMAs, including Mastery (0.591) and Enjoyment (0.477), play a pivotal role in sustaining practitioners’ commitment to their training. This finding aligns with Ryan et al. (1997) research, which also identified that persistence among Tae Kwon Do participants was significantly associated with enjoyment and competence motives, underscoring the universal importance of intrinsic motivations in the continuity of martial arts practice.

The significant factor loading of Mastery (0.591) reveals that the drive to enhance skills and competencies is a crucial motivator for individuals to continue their CMAs practice. This motivation highlights the practitioners’ dedication to personal improvement and a profound engagement with CMAs as both an art and a discipline. It reflects a deep-seated interest in mastering intricate skills and gaining an in-depth understanding of CMAs culture. Chu et al. (2009) and Ryan et al. (1997) have highlighted the significance of Mastery motivation - defined as the drive to acquire new skills and enhance personal abilities - in fostering persistence in exercise routines. This motivation for mastery, underscored by both studies, is validated within the context of this investigation, particularly among practitioners of CMAs.

The narrative shared by participants vividly illustrates how the motivation for Mastery underpins the persistence in practicing CMAs. Participants exhibited a deep fascination with the complex technical frameworks, historical evolution, and combat techniques of CMAs, reflecting a commitment that transcends mere cultural appreciation to include a genuine desire for personal growth and self-improvement. For instance, one respondent’s pursuit of martial arts styles’ “old flavor” exemplifies a passion that extends beyond physical practice to a comprehensive exploration of the culture and history that underlie CMAs. This individual’s dedication to learning Chinese characters to interpret ancient texts demonstrates an endeavor to gain a nuanced understanding of the practices, highlighting a search for traditional and cultural roots that signify a profound respect and connection with martial arts beyond mere physicality. This inclination towards understanding the foundational philosophies and historical context of martial arts, as highlighted in the study by Jones et al. (2006), underscores the depth of engagement with CMAs that goes beyond the surface level of physical skills to embrace a holistic appreciation of its cultural significance and personal relevance. Additionally, another participant’s fascination with the combat techniques of Shaolin Kung Fu signals a preference for technical mastery and practical application. Conversely, a different respondent expressed a deep emotional bond and identification with learning CMAs, describing each movement and technique as a ‘gift’. This perspective reveals a journey of not just acquiring combat skills but also learning valuable life lessons in self-respect, discipline, and resilience, thus portraying it as a comprehensive life lesson. In essence, these examples underscore the pivotal role of Mastery motivation in fostering a sustained commitment to CMAs.

Transitioning from the profound influence of Mastery motivation on the persistence of CMAs practice, it’s equally important to consider the role of Enjoyment motivation. The high scoring of Enjoyment (M = 6.343) among the motivational factors underscores the intrinsic joy and satisfaction derived from practicing CMAs. This enjoyment is not merely ancillary; as corroborated by prior research, the pleasure experienced during physical activities serves as a significant determinant for the continuation of such practices (Ryan et al., 1997; Lee, 2018; Rodrigues et al., 2020). This study further affirms the vital role of enjoyment in enhancing practitioners’ dedication to CMAs, illustrating how the pleasure gained from practice effectively promotes persistence.

In summary, the combination of Mastery and Enjoyment motivations not only deepens practitioners’ understanding and identification with CMAs culture but also increases the pleasure and personal satisfaction of practicing CMAs, thus positively affecting the persistence in practicing CMAs. These findings emphasize the importance of focusing on and cultivating intrinsic motivations in the promotion of CMAs, especially in international dissemination, by reinforcing CMAs as a means of skill enhancement and pleasure, to increase audience participation and persistence.

##### 4.2.1.2 Extrinsic psychophysical motivation

Extrinsic psychophysical motivations, encompassing Physical Condition, Psychological Condition, and Appearance, significantly contribute to the persistence in practicing CMAs, as evidenced by standardized factor loadings of Physical Condition (0.835), Psychological Condition (0.654), and Appearance (0.691). These loadings underscore the critical role these motivations play for CMAs practitioners. Initially, the predominant role of Physical Condition reflects the practitioners’ acknowledgment of CMAs as a potent physical exercise modality. The health advantages of CMAs, extensively documented and affirmed within the international academic community, have garnered recognition from worldwide health organizations, attracting a global cohort of participants (Cao and Lin, 2020). This has led to a surge in individuals engaging in CMAs to attain enhanced physical health and a more robust physique.

The substantial factor loading of Psychological Condition motivation reveals a dual focus of CMAs practice, aimed not only at physical health enhancement but also at bolstering psychological well-being. Ways of martial arts include certain forms of psychophysical culture, which, based on the tradition of warrior cultures, lead through training in fighting techniques to psychophysical improvement and self-realization (Cynarski, 2017), emphasizing the holistic development achieved through CMAs. Practices, especially those centered around internal regulation and meditation like Tai Chi, are reputed to mitigate stress, bolster emotional stability, and fortify psychological resilience (Wang et al., 2014). Furthermore, complementing these psychological benefits, research by Twemlow and Sacco (1998) highlights another dimension of CMAs’ impact on psychological conditioning. It demonstrates how traditional CMAs training significantly contributes to reducing violent tendencies in adolescents. This effect is linked to the training’s focus on promoting self-control and nurturing respect for others, illustrating CMAs’ practice as a comprehensive development of both moral and spiritual qualities, beyond mere physical activity.

Additionally, the significant factor loading attributed to Appearance motivation reflects a prevalent pursuit among the international audience to refine their physique and appearance through CMAs. In contemporary society, an appealing appearance and a healthy physique are often seen as symbols of personal image and confidence. The proliferation of social media has made this phenomenon even more pronounced, expanding the influence of health and aesthetic standards and intensifying appearance anxieties (Hawes et al., 2020; Deng and Jiang, 2023). Against this backdrop, CMAs, as a comprehensive exercise regimen, effectively help practitioners enhance their physical beauty and overall health. This focus on appearance likely mirrors the prevailing health and aesthetic standards in modern society, showcasing CMAs’ ability to fulfill individuals’ desires for an attractive physique along with health benefits.

In conclusion, the triad of extrinsic psychophysical motivations – Physical Condition, Psychological Condition, and Appearance - has a positive impact on the sustained engagement in CMAs practice. These factors not only bolster the physical and psychological well-being of practitioners but also cater to their aspirations for an improved appearance, thereby reinforcing their commitment and continuity in CMAs practice. The research findings by Jiang and Wu (2004) corroborate this observation, highlighting the crucial role of these motivational factors in fostering long-term exercise adherence. These insights hold significant implications for the worldwide propagation of CMAs, advocating for promotional strategies that emphasize the multifaceted benefits of CMAs in enhancing physical health, psychological well-being, and appearance.

##### 4.2.1.3 Extrinsic social motivation

Extrinsic social motivations - Affiliation, Competition/Ego, and Others’ Expectations - do not exhibit a significant correlation with the persistence of practitioners in CMAs. While Affiliation is typically considered a key motivator in various sports activities, its influence on the continuity of CMAs practice is minimal. A plausible explanation for this lies in the intrinsic values embedded in CMAs, particularly the ethic of Nature and Man, which fundamentally shapes the practitioner’s approach to CMAs. The ethic of Nature and Man, fundamental to traditional CMAs, promotes harmony between humans and nature, fostering independence by encouraging practitioners to value personal insight and resilience over external influences. This philosophy also drives the pursuit of self-improvement and skill mastery, explaining why Affiliation has little impact on the commitment to CMAs practice. Through fostering a strong internal motivation for personal growth, it underlines the importance of self-development in sustaining practice (Li and Guo, 2016). The ethos within CMAs culture, encapsulated by the adage “The master introduces the path, practice is personal,” underscores the emphasis on individual commitment and internal enlightenment. This ethos suggests that beyond a certain proficiency level, practitioners are encouraged to internalize and personally refine their skills. Consequently, those with a penchant for solitude and self-motivation might find themselves more aligned with the demands and rewards of CMAs practice.

Furthermore, the Competition/Ego motivation, typically linked to aspirations for superiority and success in competitive sports (Witkowski et al., 2013), has a negligible impact on the dedication to CMAs practice. This observation may indicate that CMAs are internationally recognized more as a medium for cultural engagement and self-improvement rather than as a competitive endeavor. This finding contrasts with the research by Witkowski et al. (2013) and Molanorouzi et al. (2015), where Witkowski et al. noted that judokas often prioritize winning prestigious competitions, and Molanorouzi et al. identified competition/ego as the most significant motivational factor for participation in martial arts like karate, taekwondo, and tai chi. This discrepancy raises interesting questions about the role of competition as a motivational factor in martial arts practice. A possible explanation for the diminished role of competition/ego in our study could be related to the specific styles of martial arts examined. There’s a distinction between practitioners of combat sports, such as Judo, and those of form-based martial arts, like Tai Chi. This difference stems from the focus in non-contact disciplines on self-control and self-fulfillment, rather than exclusively on the efficacy of combat. Additionally, this difference may also be related to the cultural contexts underlying these practices. CMAs, with their rich historical and philosophical underpinnings, may attract practitioners with different sets of motivations compared to those drawn to martial arts for competitive success. In particular, the emphasis in many CMAs on internal development, mindfulness, and the cultivation of Qi (vital energy) may appeal more to those seeking personal growth and health benefits than to those motivated by competition. Moreover, the cultural values associated with CMAs, such as harmony, respect, and self-discipline, might further de-emphasize the importance of competition, highlighting the art’s role in personal and spiritual development. In contrast, martial arts like karate and taekwondo, though they also embody deep cultural and philosophical principles, might be more closely associated with the competitive sport aspect in international perceptions, thereby attracting practitioners with a stronger orientation towards competition/ego. In conclusion, the variance in motivational factors between this study and that of Molanorouzi et al. (2015) underscores the complexity of martial arts as a global phenomenon and the need to consider the cultural and stylistic nuances when examining practitioners’ motivations. It suggests that the pursuit of martial arts, particularly CMAs, transcends the mere desire for competitive success, embodying a deeper search for cultural connection and personal refinement.

Lastly, while the expectation of others may initially motivate some individuals to engage in CMAs, this study reveals that such external pressures have a minimal effect on long-term commitment to CMAs practice. This finding suggests that the sustained practice of CMAs relies more on intrinsic motivation and personal commitment rather than external validation or expectations. This emphasizes the importance of recognizing the unique motivational dynamics that influence persistence in CMAs, suggesting a need for future promotional and educational strategies to highlight the personal and cultural benefits of CMAs practice, tailored to foster long-term engagement.

Overall, the combined influence of motivational factors such as Enjoyment, Mastery, Physical Condition, Psychological Condition, and Appearance forms a multifaceted motivational framework that significantly impacts the persistence of practitioners in CMAs (*β* = 0.297, *p* < 0.01). This composite motivation structure underscores that practitioners’ dedication to CMAs is not fueled by a singular motive but rather by a synergistic effect of various motivators. This insight underlines the necessity of acknowledging the multifaceted nature of motivation in enhancing the persistence of CMAs practice, especially when promoting CMAs. In the context of global dissemination, it becomes imperative to enrich practitioners’ experiences and perceptions related to skill advancement, inner satisfaction, health benefits, psychological welfare, and appearance enhancement.

#### 4.2.2 The impact of Sense of Belonging on Persistence in Practicing CMAs

Sense of Belonging embodies an individual’s perception of affiliation with an organization, distinguishing itself from the affiliation motive in CMAs practice. Whereas affiliation involves engaging in CMAs to foster social connections, Sense of Belonging encapsulates the experience of affiliation felt within a group. This concept is evaluated through six indicators, whose standardized factor loadings are as follows: “Other Kung Fu brothers and sisters here like me the way I am” (0.888), “Other Kung Fu brothers and sisters in my group respect me” (0.835), “Other Kung Fu brothers and sisters in my group take my opinions seriously” (0.776), “People in my group are friendly to me” (0.773), “I can truly be myself in this group” (0.754), “Others in the group notice when I’m skilled at something” (0.681). These metrics collectively underscore a pivotal theme: practitioners’ desire for acceptance, respect, and validation of their personal worth within the CMAs community.

Our findings demonstrate that a strong Sense of Belonging significantly influences the continuity of CMAs practice (*β* = 0.268, *p* < 0.01). Specifically, a heightened sense of organizational belonging is directly linked to increased perseverance in CMAs, aligning with the outcomes of prior research (Carron et al., 1988). This correlation suggests that the support and respect from peers within the CMAs community are crucial factors in bolstering practitioners’ dedication to CMAs. Similarly, a study conducted in Melbourne, Australia, found that a Sense of Belonging (*β* = 0.644, *p* < 0.05) is one of the key predictors in attracting and retaining members in martial arts clubs, further corroborating our findings that an enhanced sense of organizational belonging is crucial for increasing perseverance in CMAs (Kopanidis, 2023).

Moreover, Sense of Belonging amplifies practitioners’ sense of identity and role within their community, thereby fostering deeper commitment and sustained participation in CMAs activities. In Chinese martial arts, which are deeply embedded in cultural traditions, the mentor-disciple relationship is essential in nurturing a Sense of Belonging among practitioners. This dynamic, as highlighted by Levett-Jones et al. (2009), is a key influence on students’ feelings of connectedness. The Initiation Ceremony, an integral tradition within this relationship, plays a significant role in strengthening communal ties and shaping a collective identity within the CMA community.

The Initiation Ceremony is characterized by its profound ritualistic elements, beginning with incense offerings to heaven, earth, and the ancestral masters. This act symbolizes respect and the formal acceptance of a new disciple. Following this, the disciple’s bows and kowtows to the mentor, adhering to the “three bows and nine kowtows” tradition, further cement the disciple’s commitment and reverence. The exchange of the initiation certificate during the ceremony marks the disciple’s formal induction into the lineage. The culmination of this ceremony with the offering of tea and the hosting of a banquet by the disciple in honor of the mentor celebrates the establishment of the mentor-disciple bond (Xing and Zhou, 2013).

Participants in the Initiation Ceremony experience a deep moral and ceremonial sense, which transcends mere external formality to reinforce value identification, such as respect for the mentor and commitment to the martial path, fostering trust and unity within the community. This ceremony not only integrates disciples into the lineage, providing them with a Sense of Belonging, responsibility, and mission but also enhances their cultural identity and commitment to the practice. Additionally, CMA schools often honor their founding masters through portraits, and mentors lead disciples in respectful salutations, instilling a sense of reverence and transforming external rituals into internal awareness, which promotes self-affirmation and strengthens the collective identity of the practitioners within their moral community (Lin and Cao, 2020).

Reflecting on Durkheim (2016), the concept that a group bonded by shared values, behavioral norms, or rituals forms a “moral community” is highlighted. This notion is supported by Jennings et al. (2010), who view the martial arts school as a “moral community” akin to a secular religion, where beliefs and practices converge to forge a strong communal identity among practitioners. One respondent vividly described Chinese Martial Arts as providing “meaning or belonging; it’s something I can put my mind to, and the more I learn, the more I can learn,” encapsulating the profound personal and communal significance of CMAs.

In essence, the Sense of Belonging is integral to sustaining practice within CMAs. The establishment of mentor-disciple relationships, the ritualistic aspects of the Initiation Ceremony, and the formation of a moral community all contribute to a deep Sense of Belonging among practitioners. This belonging not only solidifies their cultural identity but also fuels their continuous passion and dedication to the practice. Thus, in promoting CMAs on an international scale, it is essential to emphasize the importance of these cultural and ritualistic elements to enhance practitioners’ Sense of Belonging and ensure their long-term commitment and persistence in the art.

### 4.3 Limitations and directions for future research

This study aimed to explore the impact of motivations for practicing CMAs on the persistence of such practice and employed a second-order model to aggregate motivation factors significantly associated with persistence (such as enjoyment, mastery, physical and psychological health, and appearance) to understand how these motivations collectively influence the persistence in practicing CMAs. However, this research did not individually explore the differences in the impact of these motivation factors on the persistence of practicing CMAs, necessitating further detailed examination of their specific contributions to persistence in future studies.

Moreover, while this study included 226 participants from various countries and age groups, it did not delve into the potential impact of participants’ background characteristics on the research model. Research by Ding et al. (2015) found significant differences in the motivations of CMAs practitioners based on gender, skill level, martial arts style, and membership type. Zeng (2019) further noted that while nationality showed variations in participation motivation, skill level was not a decisive factor. In Ding et al. (2015) study, comparisons were made between the novice group and the advanced group, whereas Zeng (2019) focused on the comparison between medium level and high level. Importantly, in specific traditional CMAs styles, due to the lack of competition and grading/ranking systems, determining a Chinese martial artist’s skill level is often challenging. These findings suggest that future research should conduct more detailed analyses of background variables such as gender, age, nationality, and martial arts style to explore how these factors influence the motivation to persistently practice CMAs, providing a comprehensive view of the complex dynamics behind different practitioners’ persistence in practicing CMAs.

Lastly, this study accounted for only 22.1% of the variance in Persistence in Practicing CMAs, indicating that other important factors not explored in this research could influence persistence. Therefore, future research could investigate additional variables, such as personal goals, social support, and the interaction between instructors and practitioners, to provide a more comprehensive understanding of the motivation to persistently practice CMAs. By exploring these factors, future research can further promote a deeper understanding of sustained engagement in CMAs practice, thereby contributing to the global dissemination and cultural exchange of CMAs more effectively.

## Data availability statement

The raw data supporting the conclusions of this article will be made available by the authors, without undue reservation.

## Ethics statement

Ethical approval was not required for the studies involving humans because this study involved non-sensitive, anonymous data collection through public domain research methods, with no potential risk to participants. The studies were conducted in accordance with the local legislation and institutional requirements. Written informed consent for participation was not required from the participants or the participants’ legal guardians/next of kin in accordance with the national legislation and institutional requirements because implied consent was obtained through completion of the survey, in line with the consent statement provided at the beginning of the survey.

## Author contributions

XC: Conceptualization, Data curation, Formal analysis, Funding acquisition, Investigation, Writing – original draft, Writing – review & editing. HL: Investigation, Methodology, Writing – review & editing.
