# Supplementary material for: Motivational drivers and Sense of Belonging: unpacking the persistence in Chinese Martial Arts practice among international practitioners
Source: Front Psychol. 2024 May 2;15:1403327. doi: 10.3389/fpsyg.2024.1403327 (PMC11097903; doi:10.3389/fpsyg.2024.1403327)
Supplement: Supplementary file 1 [file Table_1.DOCX]

Supplementary Material

# Supplementary Figures and Tables

| **Factor** | **Reference** | **Item No.** | **Item** |
| --- | --- | --- | --- |
| **Enjoyment** | Morris and Rogers (2004)  Molanorouzi et al. (2014) | Q14a_1 | Because it’s interesting. |
|  |  | Q14a_2 | Because it makes me happy. |
|  |  | Q14a_3 | Because it’s fun. |
|  |  | Q14a_4 | Because I enjoy exercising.* |
|  |  | Q14a_5 | Because I have a good time. |
| **Mastery** |  | Q14b_1 | To get better at Chinese martial arts.* |
|  |  | Q14b_2 | To improve existing skills. |
|  |  | Q14b_3 | To do my personal best. |
|  |  | Q14b_4 | To obtain new skills/activities. |
|  |  | Q14b_5 | To keep current skill level.* |
| **Affiliation** |  | Q14c_1 | Because I enjoy spending time with others.* |
|  |  | Q14c_2 | To do it with others. |
|  |  | Q14c_3 | To do something in common with friends. |
|  |  | Q14c_4 | To talk with friends exercising. |
|  |  | Q14c_5 | To be with friends. |
| **Physical condition** |  | Q14d_1 | Because it helps maintain a healthy body. |
|  |  | Q14d_2 | Be physically fit. |
|  |  | Q14d_3 | To maintain physical health.* |
|  |  | Q14d_4 | Because it keeps me healthy.* |
|  |  | Q14d_5 | To improve cardiovascular fitness. |
| **Competition/Ego** |  | Q14e_1 | Because I perform better than others. |
|  |  | Q14e_2 | To be best in the group.* |
|  |  | Q14e_3 | To work harder than others. |
|  |  | Q14e_4 | To compete with others around me. |
|  |  | Q14e_5 | To be fitter than others. |
| **Appearance** |  | Q14f_1 | To define muscle, look better. |
|  |  | Q14f_2 | To improve body shape. |
|  |  | Q14f_3 | To improve appearance.* |
|  |  | Q14f_4 | To lose weight, look better. |
|  |  | Q14f_5 | To maintain trim, toned body. |
| **Psychological condition** |  | Q14g_1 | Because it helps me relax. |
|  |  | Q14g_2 | To better cope with stress. |
|  |  | Q14g_3 | To get away from pressures. |
|  |  | Q14g_4 | Because it acts as a stress release. |
|  |  | Q14g_5 | To take mind off other things.* |
| **Others’ expectations** |  | Q14h_1 | To earn a living.* |
|  |  | Q14h_2 | Because I get paid to do it. |
|  |  | Q14h_3 | To manage medical condition.* |
|  |  | Q14h_4 | Because people tell me I need to. |
|  |  | Q14h_5 | Because it was prescribed by doctor, physio. |
| **Sense of belonging** | Allen (2006) | Q13_1 | I feel like a part of my group.* |
|  |  | Q13_2 | Other Kung Fu brothers and sisters in my group take my opinions seriously. |
|  |  | Q13_3 | I am included in many of the group activities.* |
|  |  | Q13_4 | I can really be myself on this group. |
|  |  | Q13_5 | Other Kung Fu brothers and sisters here like me the way I am. |
|  |  | Q13_6 | People in my group are friendly to me. |
|  |  | Q13_7 | Others in the group notice when I’m good at something. |
|  |  | Q13_8 | I am treated with as much respect as others.* |
|  |  | Q13_9 | People know I can perform well.* |
|  |  | Q13_10 | I feel proud of belonging to this group.* |
|  |  | Q13_11 | Other Kung Fu brothers and sisters in my group respect me. |
| **PiPCMAs** | Liu et al. (2011) | Q8_1 | Practicing Chinese martial arts has become a habit for me.* |
|  |  | Q8_2 | Despite my limited time, I've never stopped practicing Chinese martial arts. |
|  |  | Q8_3 | I would feel disheartened if I had to stop practicing Chinese martial arts. |
|  |  | Q8_4 | Living without Chinese martial arts would be difficult for me. |
|  |  | Q8_5 | Even though there are difficulties of practicing Chinese martial arts, I am willing to insist on practicing it. |
|  |  | Q8_6 | I always eagerly anticipate my next martial arts practice session.* |

**Supplementary Table 1.** Overview of Scale Utilization. * items removed during the measurement model fitting process.
